# Successful *i*-GONAD in Mice at Early Zygote Stage through In Vivo Electroporation Three Min after Intraoviductal Instillation of CRISPR-Ribonucleoprotein

**DOI:** 10.3390/ijms231810678

**Published:** 2022-09-14

**Authors:** Shuji Takabayashi, Kenta Iijima, Masumi Tsujimura, Takuya Aoshima, Hisayoshi Takagi, Kazushi Aoto, Masahiro Sato

**Affiliations:** 1 Laboratory Animal Facilities & Services, Preeminent Medical Photonics Education & Research Center, Hamamatsu University School of Medicine, 1-20-1 Handayama, Higashi-ku, Hamamatsu, Shizuoka 431-3192, Japan; 2 Department of Biochemistry, Hamamatsu University School of Medicine, 1-20-1 Handayama, Higashi-ku, Hamamatsu, Shizuoka 431-3192, Japan; 3 Department of Genome Medicine, National Center for Child Health and Development, Tokyo 157-8535, Japan

**Keywords:** *i*-GONAD, in vivo electroporation, genome editing, early zygotes, oviduct, cumulus cells, indels, hyaluronidase

## Abstract

Improved genome editing via oviductal nucleic acids delivery (*i*-GONAD) is a new technology enabling in situ genome editing of mammalian zygotes exiting the oviductal lumen, which is now available in mice, rats, and hamsters. In this method, CRISPR/Cas9 genome-editing reagents are delivered directly to the oviducts of pregnant animals (corresponding to late zygote stage). After intraoviductal instillation, electric shock to the entire oviduct was provided with a specialized electroporation (EP) device to drive the genome editing reagents into the zygotes present in the oviductal lumen. *i*-GONAD toward early zygotes has been recognized as difficult, because they are tightly surrounded by a cumulus cell layer, which often hampers effective transfer of nucleic acids to zygotes. However, in vivo EP three min after intraoviductal instillation of the genome-editing reagents enabled genome editing of early zygotes with an efficiency of 70%, which was in contrast with the rate of 18% when in vivo EP was performed immediately after intraoviductal instillation at Day 0.5 of pregnancy (corresponding to 13:00–13:30 p.m. on the day when vaginal plug was recognized after natural mating). We also found that addition of hyaluronidase, an enzyme capable of removing cumulus cells from a zygote, slightly enhanced the efficiency of genome editing in early zygotes. These findings suggest that cumulus cells surrounding a zygote can be a barrier for efficient generation of genome-edited mouse embryos and indicate that a three-minute interval before in vivo EP is effective for achieving *i*-GONAD-mediated genome editing at the early zygote stage. These results are particularly beneficial for researchers who want to perform genome editing experiments targeting early zygotes.

## 1. Introduction

Recent advances in the clustered regularly interspaced short palindromic repeat-associated protein 9 (CRISPR/Cas9)-mediated gene-editing system has made it possible to modify the genome of various organisms. Laboratory mice and rats have been widely used as common models of human diseases; however, the current standard method to create genome-engineered animals involves three major steps: isolation of zygotes from females, ex vivo handling of zygotes (i.e., microinjection into zygotes, electroporation (EP) using a specialized EP device and cultivation of genome-engineered zygotes) and egg transfer (ET) of the genome-engineered embryos into pseudopregnant females to develop them further [1,2,3,4,5]. These steps are costly, laborious and time-consuming and often require persons with a special technique (to manipulate a micromanipulator and perform ET). To bypass this ex vivo handling process of zygotes, we recently developed a novel method (called “improved genome editing via oviductal nucleic acids delivery, *i*-GONAD”) which relies on in situ genome editing of zygotes present in the oviductal lumen of a pregnant female [6]. In this method, CRISPR/Cas9 genome-editing reagents are delivered directly to the oviducts of pregnant animals (corresponding to late zygote stage). Immediately after intraoviductal instillation, electric shock to the entire oviduct was provided with a specialized EP device to drive the genome-editing reagents into the zygotes present in the oviductal lumen. According to Ohtsuka et al. [6], insertion or deletion of nucleotides (indels) was induced at a target locus (forkhead box protein E3 gene, *Foxe3*) with efficiencies of 97%. In addition, targeted knock-in (KI) of a sequence into a target locus (tyrosinase gene, *Tyr*) occurs with efficiencies of 50%, when a solution containing Cas9 protein, single guide RNA (sgRNA) and a donor sequence to be knocked-in was introduced. 

Notably, late zygotes are generally present in the ampulla of an oviduct of a female mouse at Day 0.7 of pregnancy [7,8]. This stage roughly corresponds to 16:00~17:00 p.m. on the day when vaginal plug was recognized. The presence of a vaginal plug at noon was designated as Day 0.5 of pregnancy. Late zygotes are almost free of cumulus cells, whereas early zygotes, existing in the ampulla portion of an oviduct at Day 0.4 of pregnancy (corresponding to 10:00~11:00 a.m.), are tightly surrounded by a cumulus cell layer [6]. Our previous attempt to transfect early zygotes with nucleic acids in vivo failed [6,9,10]. We considered that this failure is ascribed to the presence of a cumulus cell layer that might have hampered efficient transfer of nucleic acids to zygotes even in an environment where electric shock to drive nucleic acids to deliver to embryos is provided by an EP device. One idea to overcome this problem may be that early zygotes are pretreated with hyaluronidase (HA), an enzyme capable of dispersing cumulus cells from a zygote [7], prior to *i*-GONAD. Kaneko and Tanaka [11] examined the possibility by injecting 1 μL of 0.1% HA into the ampulla of a female (ICR) at Day 0.4 of pregnancy using a thin glass needle. As a control, the solvent (phosphate-buffered saline, PBS) was similarly injected. Several minutes after the injection, a solution (1 μL) containing genome editing reagents (2 μg/μL Cas9 protein + 60 mM dual gRNA (targeted fibroblast growth factor 10 gene (*Fgf10*) + 0.08% trypan blue) was intraoviductally introduced, and subsequently, the entire oviducts were subjected to in vivo EP using tweezer-type electrodes. After that, the developing fetal offspring were isolated for examining the presence of possible genome editing in those samples. Consequently, the samples isolated from the HA-treated group exhibited 2.5-fold higher genome editing (indels) efficiency than those isolated from the control group (68% vs. 27%). The *i*-GONAD on Day 0.7 of pregnancy (in which case no HA is used) yielded genome-edited pups with an efficiency of 54%. These findings indicate that HA-mediated removal of cumulus cells at Day 0.4 of pregnancy is effective when in situ genome editing toward early zygotes are intended. 

Besides the HA-aided removal of cumulus cells, we considered that it may require several minutes (min) for the exogenous solution injected into the oviductal lumen to spread into the cumulus cells and zygotes. This in turn implies in vivo EP several min after intraoviductal instillation will result in enhanced gene-editing efficiency in early zygotes. In this study, we aimed to achieve *i*-GONAD toward early zygotes without the use of HA. At first, we examined the possible effects of 3 min interval (vs. 0 min interval) before in vivo EP on the genome-editing efficiency of early zygotes in mice. Second, we examined whether the addition of HA to the CRISPR reagent is beneficial for improving *i*-GONAD-mediated genome editing at this stage.

## 2. Results

### 2.1. Three Min Interval before In Vivo EP Is Effective to Induce Indels in Early Zygotes

We first examined the possible effects of a 3 min interval (vs. 0 min interval) before in vivo EP on the genome-editing efficiency of early zygotes in mice. Then, we monitored how the solution injected into an ampulla of an oviduct is distributed within an oviductal lumen, and next confirmed whether *i*-GONAD under the condition of 3 min with RNP + dye can be useful for efficient generation of genome-edited early zygotes.

**Figure 1:**
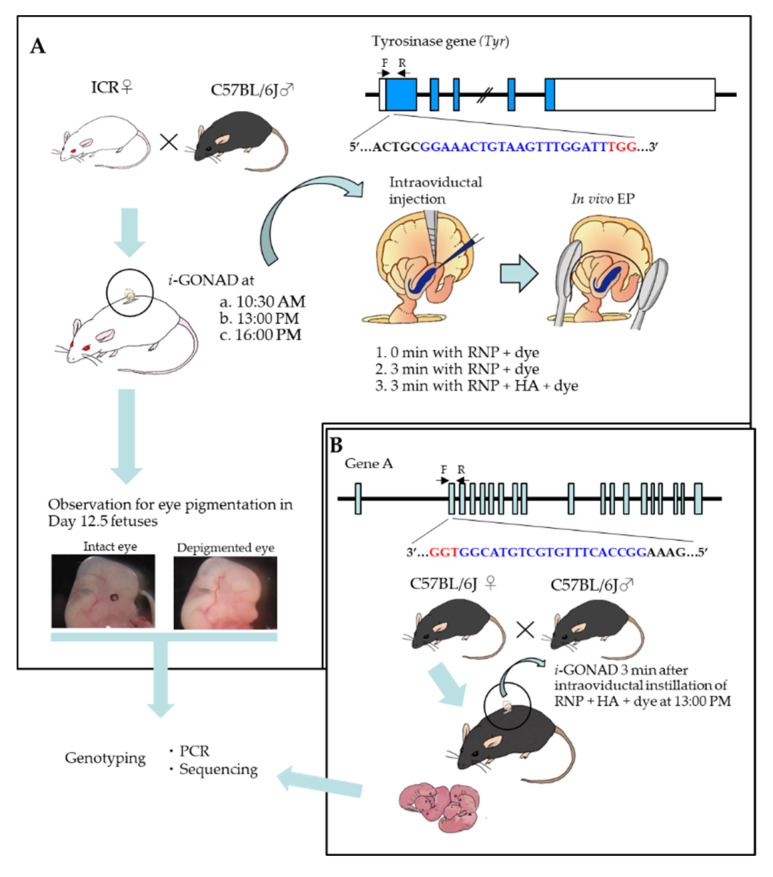
Experimental outline of *i-*GONAD performed toward zygotes at various times using different conditions and reagents. (**A**). *i-*GONAD-mediated disruption of tyrosinase gene (*Tyr*). One day before *i*-GONAD, female ICR mice are naturally mated to male C57BL/6J mice. The next day, the ICR females showing successful pregnancy, which can be judged by the presence of copulation plugs in the vagina, were subjected to *i*-GONAD at various times (10:30 a.m., 13:00 p.m. and 16:00 p.m.) using different conditions (in vivo electroporation (EP) immediately (0 min) after intraoviductal instillation of ribonucleoprotein (RNP) + Fast Green FCF (dye), which is defined as “0 min with RNP + dye”, in vivo EP 3 min after intraoviductal instillation of RNP + dye, which is defined as “3 min with RNP + dye” or in vivo EP 3 min after intraoviductal instillation of RNP + hyaluronidase (HA) + dye, which is defined as “3 min with RNP + HA + dye”. At Day 12.5 of pregnancy, mid-gestational fetuses are dissected from the *i-*GONAD-treated females to check the presence of de-pigmented eyes and subsequent genotyping. The sequence recognized by gRNA is shown in blue and protospacer adjacent motif (Pa.m.) by red. (**B**). *i-*GONAD-mediated disruption of Gene A. In a case of disruption of Gene A, in vivo *EP* was performed on the pregnant B6 females at Day 0.5 of pregnancy 3 min after intraoviductal instillation of RNP (targeted Gene A) + HA + dye. Then, the *i-*GONAD-treated females were allowed to survive for delivering their pups. The sequence recognized by gRNA is shown in blue and protospacer adjacent motif (Pa.m.) in red.

As shown in Figure 1A, two types of reagents (ribonucleoprotein (RNP) + dye or RNP + HA + dye) were introduced into the oviductal lumen of pregnant ICR females at 10:30–11:00 a.m. (hereinafter defined as 10:30 a.m.; corresponding to early zygote stage), 13:00–13:30 p.m. (hereinafter defined as 13:00 p.m.; corresponding to zygote stage showing early-to-late transition) or 16:00–16:30 p.m. (hereinafter defined as 16:00 p.m.; corresponding to late zygote stage). In the case of injection of RNP + dye, in vivo EP was performed immediately (0 min) or 3 min after intraoviductal instillation. The former treatment is defined hereinafter as “0 min with RNP + dye”, and the latter as “3 min with RNP + dye”. In the case of injection of RNP + HA + dye, in vivo EP was performed 3 min after intraoviductal instillation, which is defined hereinafter as “3 min with RNP + HA + dye”. The RNP used comprised a Cas9 protein and dual gRNA targeting tyrosinase gene (*Tyr*), which codes for proteins essential for eye pigmentation. Since the pregnant Slc:ICR (ICR) females were derived from mating between ICR females and C57BL/6JJmsSlc (B6) males (Figure 1A), their offspring (including fetuses) should have a wild-type *Tyr* allele derived from the B6 trait with pigmented eyes. Therefore, targeted disruption of *Tyr* in one allele carrying wild-type *Tyr* will lead to the generation of fetuses with de-pigmented eyes. After *i*-GONAD, the treated females were allowed to survive until Day 12 of pregnancy. Then, the fetuses were isolated for monitoring phenotypic alteration in eyes and subsequent genotyping (PCR and sequencing). 

**Figure 2:**
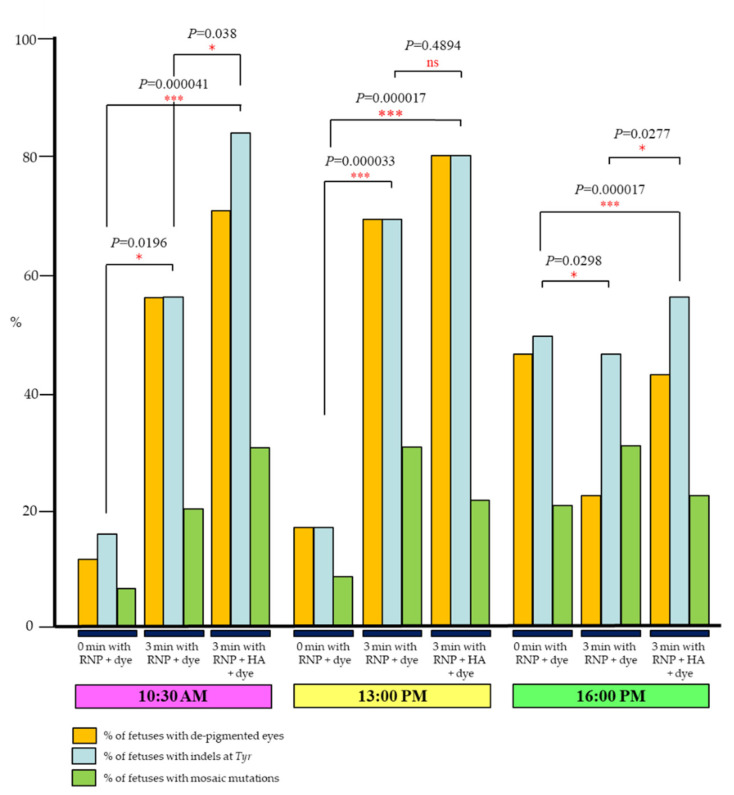
Summary of the experiments using *i*-GONAD-mediated genome editing targeting *Tyr* as shown in Figure 1A. The detailed data are shown in Table S1. *i*-GONAD was performed at various times (10:30 a.m., 13:00 p.m. and 16:00 p.m.) in the day (when vaginal plugs were first discerned) using different conditions (in vivo electroporation (EP) immediately (0 min) after intraoviductal instillation of ribonucleoprotein (RNP) + Fast Green FCF (dye), which is defined as “0 min with RNP + dye”, in vivo EP 3 min after intraoviductal instillation of RNP + dye, which is defined as “3 min with RNP + dye” or in vivo EP 3 min after intraoviductal instillation of RNP + hyaluronidase (HA) + dye, which is defined as “3 min with RNP + HA + dye”. At Day 12.5 of pregnancy, mid-gestational fetuses are dissected from the *i-*GONAD-treated females, in which *Tyr* locus had been targeted. Percent of fetuses showing de-pigmented eyes (filled orange), idels at *Tyr* (filled blue) and mosaic mutations (filled green) are shown as graphs. Statistical analysis was performed using chi-square test; * *p* < 0.05; *** *p* < 0.001; ns, not significant.

The results are shown in Figure 2 and Table S1. When *i*-GONAD was performed at 10:30 a.m., 12% (2/17) of fetuses had de-pigmented eyes in the group of 0 min with RNP + dye, while 55% (11/20) of fetuses had de-pigmented eyes in the group of 3 min with RNP + dye, which was significantly different (*p* = 0.0196). This was also confirmed by genotyping analysis: 18% (3/17) and 55% (11/20) of fetuses were identified as those with indels, when 0 min with RNP + dye or 3 min with RNP + dye was treated, respectively. Notably, pre-treatment with HA resulted in significantly (*p* = 0.038) enhanced gene editing rates (70% (14/20)) of fetuses with de-pigmented eyes as well as of those (85% (17/20)) in fetuses with indels at *Tyr* locus (“3 min RNP + HA + dye” treatment in Figure 2; Table S1). A similar tendency was also seen when *i*-GONAD was performed at 13:00 p.m.: 18% (6/33) of fetuses in the group of 0 min with RNP + dye had de-pigmented eyes, while 70% (21/30) of fetuses in the group 3 min with RNP + dye had de-pigmented eyes, which was statistically different (*p* = 0.000033) (Figure 2; Table S1). Molecular biological analysis of these fetuses confirmed the results obtained from the phenotypic analysis (Figure 2; Table S1). However, pre-treatment with HA did not significantly affect the genome editing efficiency at this stage (*p* = 0.4894). In the case of *i*-GONAD performed at 16:00 p.m., the rates of de-pigmented fetuses appeared to differ between the two groups, namely 0 min with RNP + dye and 3 min with RNP + dye (47% (21/44) vs. 22% (6/27)), but there was no statistical difference between them (*p* = 0.0298) (Figure 2; Table S1). Molecular biological analysis demonstrated that the rates of fetuses with indels did not differ between these two groups (50% (22/44) vs. 44% (12/27)) (Figure 2; Table S1). Probably, some pigmented fetuses obtained after *i*-GONAD performed under the condition of “3 min with RNP + dye” at 16:00 p.m. might have indels in the coding region of *Tyr*, although its mutation does not lead to alteration in eye pigmentation. Pre-treatment with HA (3 min with RNP + HA + dye) failed to improve the rates of de-pigmented fetuses, when compared with 3 min with RNP + dye (43% (10/23) vs. 22% (6/27), *p* = 0.0277). From these experiments, in vivo EP left for three min after intraoviductal injection with RNP + HA + dye is the most effective for inducing indels at a target locus in early zygotes. 

We next performed whether in vivo EP left for three min after intraoviductal injection with RNP + HA + dye at Day 0.5 of pregnancy (13:00 p.m.) has the ability to disrupt another endogenous locus (which is called “Gene A”; Figure 1B) with a good efficiency. We compared the genome editing efficiency in the F0 pups (derived from B6 females) obtained after *i*-GONAD under the conditions, 0 min with RNP + dye or 3 min with RNP + HA + dye on 13:00 p.m. The results are shown in Table 1. As expected, the latter treatment exhibited better genome-editing performance than the former treatment (46% (6/13) vs. 23% (3/13), *p* = 0.2162).

### 2.2. Mode of Distribution of a Solution Injected into the Oviductal Lumen

**Figure 3:**
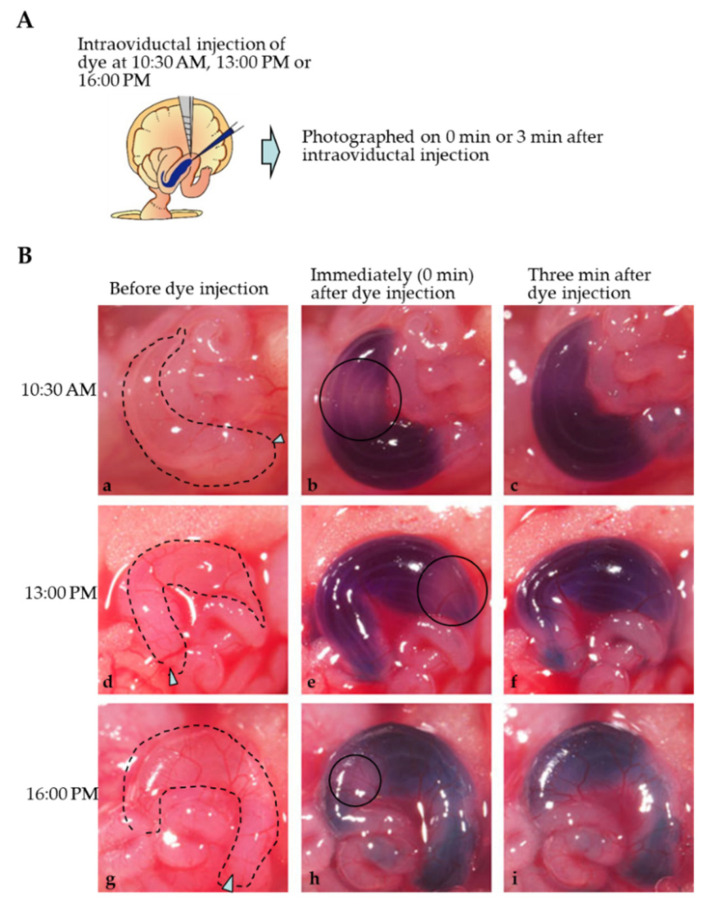
Monitoring of the solution intraoviductally injected using dye (Fast Green FCF). (**A**). Schematic representation of intraoviductal injection of dye at various times (10:30 a.m., 13:00 p.m. and 16:00 p.m.) on the day when vaginal plug are discerned for checking the mode of dye distribution at the ampulla portion immediately (0 min) or 3 min after injection. (**B**). Photographs taken before (**a**,**d**,**g**) and 0 min (**b**,**e**,**h**) or 3 min (**c**,**f**,**i**) after intraoviductal injection of dye. The ampulla portion is indicated by dotted lines in (**a**,**d**,**g**). Arrowheads in (**a**,**d**,**g**) indicate the site injected. Circles in (**b**,**e**,**h**) indicate the portions where the introduced dye is scarcely distributed and therefore looking pale. (**a**–**c**) Injection at 10:30 a.m.; (**d**–**f**) injection at 13:00 p.m.; (**g**–**i**) injection at 16:00 p.m.

Intraoviductal instillation of a solution (1–2 μL) containing 0.02% dye (Fast Green FCF) was performed at Days 0.4 (10:30 a.m.), 0.5 (13:00 p.m.) and 0.7 (16:00 p.m.) of pregnancy, and mode of dye distribution was assessed immediately (0 min after injection) and 3 min after injection, as shown schematically in Figure 3A. In the case of “0 min after injection”, distribution of the injected solution appears not to be uniform throughout an ampulla region; there are some pale areas (shown by circles in Figure 3B(b,e,h)) at each stage examined. However, these pale areas disappeared 3 min later, and the dye distribution appears to be unified throughout an ampulla region (Figure 3B(c,f,h)). These findings suggest that it takes several periods (at least 3 min) to allow the solution injected intraoviductally to be spread uniformly throughout the ampulla region.

### 2.3. Visualization of i-GONAD-Mediated Delivery of Fluorescent Reagents to Early Zygotes

**Figure 4:**
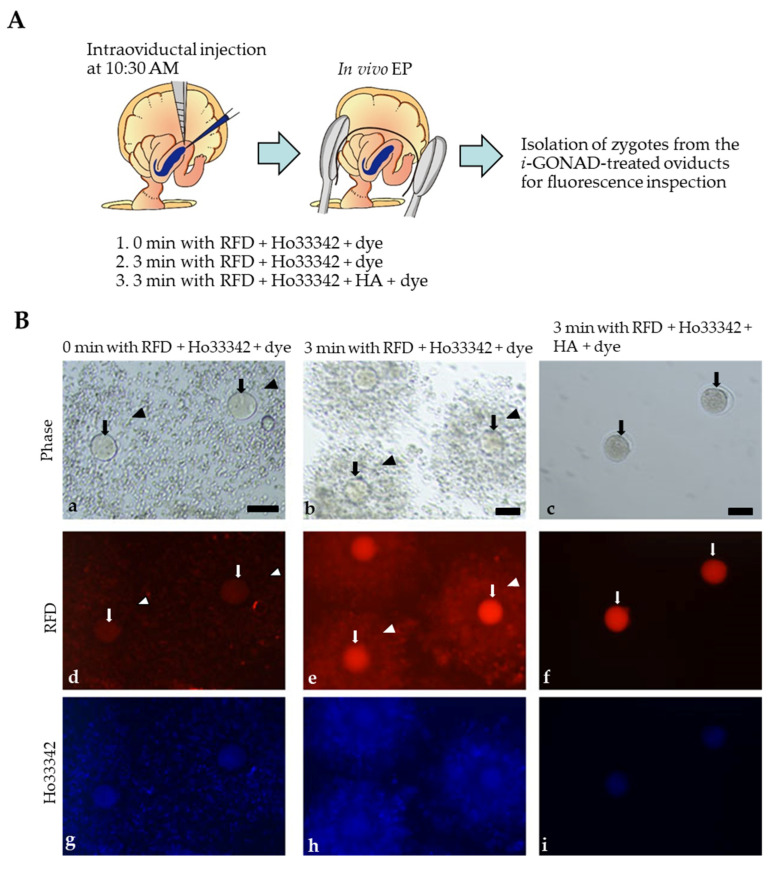
Visualization of successful *i*-GONAD at early zygote stage. (**A**). Schematic representation of intraoviductal injection of a solution containing fluorescent substances at 10:30 a.m. on the day when vaginal plug was discerned for checking possible fluorescence in zygotes and cumulus cells. In vivo EP was performed immediately (0 min) or 3 min after intraoviductal injection of a solution containing tetramethylrhodamine-conjugated dextran 3 kDa (RFD) + Hoechst 33342 (Ho33342) + Fast Green FCF (dye) (which is called “RFD + Ho33342 + dye”). In some cases, in vivo EP was performed 3 min after intraoviductal injection of a solution containing RFD + Ho33342 + hyaluronidase (HA) + dye. After in vivo EP, oviducts are dissected and subjected to isolation of a cumulus–zygote complex or cumulus cell-free zygotes for checking fluorescence. (**B**). Fluorescence inspection in the cumulus–zygote complex or cumulus cell-free zygotes isolated from the *i*-GONAD-treated females at 10:30 a.m. Note that when in vivo EP 0 min after intraoviductal injection of RFD + Ho33342 + dye was performed, no obvious RFD-derived fluorescence was discernible in zygotes (arrows in **d**), although a slight fluorescence was seen in the attached cumulus cells (arrowheads in **d**). In contrast, bright RFD-derived fluorescence was discernible in both zygotes (arrows in **e**) and cumulus cells (arrowheads in **e**) when in vivo EP 3 min after intraoviductal injection of RFD + Ho33342 + dye was performed. In vivo EP 3 min after intraoviductal injection of RFD + Ho33342 + HA + dye resulted in generation of cumulus cell-free zygotes with bright RFD-derived fluorescence (arrows in **c,f**). Bar: 100 μm.

We employed tetramethylrhodamine-labeled dextran 3 kDa (RFD) and Hoechst33342 (Ho33342) to confirm whether *i*-GONAD under the condition of 3 min with RNP + dye can be useful for efficient generation of genome-edited early zygotes. RFD is known to be useful for checking the success of *i*-GONAD within two days, because two-cell embryos obtained one day after *i*-GONAD at late zygote stage using a solution containing RFD always exhibited bright red fluorescence in their cytoplasm [6,12]. Ho33342 is a useful reagent capable of staining nucleus specifically. A solution containing RFD + Ho33342 + dye or RFD + Ho33342 + HA + dye was first subjected to intraoviductal instillation on 10:30 a.m., and then, in vivo EP was performed immediately (0 min) or 3 min after injection. After finishing in vivo EP, females were subjected to isolation of zygotes (or those with cumulus cells) for inspection of fluorescence under a fluorescence microscope. When RFD + Ho33342 + dye were injected and immediately in vivo EP was applied, no obvious RFD-derived red fluorescence was observed in zygotes (arrows in Figure 4B(d)). Cumulus cells were also slightly fluorescent (arrowheads in Figure 4B(d)). In contrast, in vivo EP 3 min after oviductal instillation resulted in generation of both zygotes (arrows in Figure 4B(e)) and cumulus cells (arrowheads in Figure 4B(e)) showing bright RFD-derived fluorescence. Notably, cumulus cells surrounding a zygote remained intact even after in vivo EP after the 3 min treatment with RFD + Ho33342 + dye (arrowheads in Figure 4B(b)), suggesting spreading of a sufficient amount of the intraoviductally injected solution into the cumulus cell layer during the 3 min treatment.

As pointed out by Kaneko and Tanaka [11], intraoviductal injection of HA results in successful removal of cumulus cells from a zygote in vivo. We confirmed this point by injecting RFD + Ho33342 + HA + dye and subsequently being left for 3 min prior to in vivo EP: the isolated zygotes were almost free of cumulus cells (arrows in Figure 4B(c)) and successfully fluorescent (arrows in Figure 4B(f)). These results suggest that a short period (3 min) of interval prior to in vivo EP after intraoviductal instillation of an RNP-containing solution is sufficient to induce *i*-GONAD-mediated genome editing at early zygote stage. 

## 3. Discussion

Early zygotes at Day 0.4 of pregnancy (10:30–11:00 a.m.) are tightly surrounded by a cumulus cell layer, which is naturally detached from a zygote as development proceeds. As a result, late zygotes at Day 0.7 of pregnancy (16:00–16:30 p.m.) are almost free from cumulus cells [6]. Our previous experiments have demonstrated that *i*-GONAD-mediated gene editing at Day 0.4 of pregnancy was more difficult than that on Day 0.7 of pregnancy [6]. We speculated that the cumulus cell layer at Day 0.4 of pregnancy may prevent efficient delivery of exogenous nucleic acids to zona pellucida-enclosed zygotes. This hypothesis was proven by Kaneko and Tanaka [11] who used HA for dissociation of the cumulus cell layer at Day 0.4 of pregnancy. They intraoviductally instilled a solution containing HA and RNP into the pregnant females at Day 0.4 of pregnancy, left for several min and then subsequently performed in vivo EP toward the injected oviducts. Consequently, genome editing efficiency in the target locus (*Fgf10*) was greatly improved when compared with intraoviductal instillation of a solution containing RNP alone and subsequent in vivo EP (27% vs. 68%). In this study, we found that a 3 min interval prior to in vivo EP is sufficient to induce indels at a target locus even in the absence of HA. This was beyond our initial hypothesis, because the cumulus cell layer at Day 0.4 of pregnancy is so tightly packed, making it difficult to infiltrate any of soluble substances into these layers even after being left for several min after oviductal instillation. This delay in filtration may often cause reduced frequency for genome editing at a target locus. However, additional experiments using a solution containing RFD + Ho33342 + dye support our present results. In vivo EP immediately (0 min) after intraoviductal instillation of the solution resulted in failure of RFD-derived fluorescence in zygotes (see arrows in Figure 4B(d)), while in vivo EP 3 min after intraoviductal instillation led to generation of fluorescent zygotes (see arrows in Figure 4B(e)). Notably, there was no sign for dissociation of cumulus cells when in vivo EP was performed 3 min after the injection (see arrowheads in Figure 4B(b)), suggesting that the above event (appearance of fluorescent zygotes) was not related to the dissociation of the cumulus cell layer. In contrast, HA treatment led to removal of cumulus cells from an early zygote (see arrows in Figure 4B(c)). Furthermore, these zygotes were fluorescent (see arrows in Figure 4B(f)). 

What happens during the 3 min interval prior to in vivo EP after intraoviductal instillation? The first point to be noted is that gradual dispersion of a solution throughout the oviductal lumen occurs during the period. Consecutive observation demonstrated that the introduced dye was not uniformly distributed immediately after dye introduction. For example, some portions were often paler than the other portion. However, 3 min after instillation, the pale area disappeared, and then, a uniform distribution of dye was visible throughout the ampulla (see the area enclosed by circles in Figure 3B). This uniform distribution of dye may be caused not only by simple dispersion of the dye, but also by peristaltic movement of the oviduct itself. Close inspection of an oviduct demonstrated that the cumulus cell–zygote complex moved slightly up and down within the oviductal lumen, suggesting that intraoviductal fluid is not static, but rather motive. The second point to be noted is the possibility that the intercellular space of cumulus cells at Day 0.4 of pregnancy is loose, which will facilitate rapid infiltration of the solution provided exogenously. 

As mentioned above, to induce *i*-GONAD-mediated genome editing in early zygotes, we must wait three min after intraoviductal injection of genome-editing reagents. In this case, serious concern may arise that oviducts placed outside the skin for a short period (only 3 min) may affect the function of the oviduct itself. We always took care that the exposed oviducts are covered with a wet paper during the operation to prevent possible evaporation from the surface of an oviduct. As a result, we successfully obtained viable pups after *i*-GONAD at Day 0.5 of pregnancy (see Table 1). 

For an attempt to increase the genome editing efficiency of early zygotes, it may be preferable to employ the following modified protocol: in vivo EP 3 min after intraoviductal instillation of a solution containing HA + RNP + dye. The addition of HA in the solution will increase the genome editing efficiency significantly in comparison with using the HA-free solution at the early zygote stage (see Figure 2 and Table S1). Notably, the indel efficiency achieved by the *i*-GONAD using the modified protocol at Day 0.4 of pregnancy (10:30 a.m.) tends to be higher than that achieved at Day 0.7 of pregnancy (85% vs. 57%; see Figure 2 and Table S1). In this context, *i*-GONAD at Day 0.4 of pregnancy may be preferable than that at Day 0.7 of pregnancy in view of acquiring genome-edited animals with high efficiency. In this study, we did not test the possibility of KI at Day 0.4 of pregnancy using the modified protocol, which will be a future subject. 

According to Kaneko and Tanaka [11], the operation time for introducing genome-editing reagents into embryos in the oviducts can be adjusted by treatment with HA before EP. This improved protocol can also be used for efficient production of genome-edited mice and rats.

## 4. Materials and Methods

### 4.1. Animals

B6 and ICR mice were purchased from Japan SLC, Inc. (Shizuoka, Japan). Adult animals at the age ranges of 8–10 weeks (female) and 10–12 weeks (male) were used. All animals were maintained under temperature-controlled conditions (24 ± 2 °C) with a 12L/12D light cycle (lights on at 7:00 a.m.). A solid diet and water were provided ad libitum. The experiments described were performed in accordance with the guidelines of *Hamamatsu University School of Medicine Committee on Recombinant DNA Security* (no. 29-17, no. 2-24). Additionally, the experiments were approved by *Hamamatsu University School of Medicine Animal Care and Use Committee* (nos. 2017097 and 2018026). The experiments involving in vivo transfection of mouse preimplantation embryos by *i*-GONAD were accompanied by surgery (exposure of ovary/oviducts/uterus) and operation/manipulation (nucleic acid injection via the oviductal wall and in vivo EP). All efforts were made to minimize the number of animals used and their suffering.

### 4.2. Mating Protocol

For mating, ICR females exhibiting signs for successful mating after visual observation of their vagina were selected and mated to B6 males. In some cases, B6 females were mated to B6 males. Each female mouse was coupled with a male in a cage in the evening. The following morning, females were checked for the presence of a vaginal plug to confirm that mating had occurred. The plugged females were subjected to *i*-GONAD. 

### 4.3. Preparation of Solutions Used for i-GONAD

For inducing the CRISPR/Cas9-mediated indels, Alt-R^®^ CRISPR-Cas9 crRNAs were designed to recognize the target site (exon 1 of *Tyr* gene (Figure 1A), a gene responsible for pigmentation, or Gene A (Figure 1B)) that matches a 20 bp DNA sequence (Tyr crRNA: GGAAACTGTAAGTTTGGATT and Gene A crRNA: GGCCACTTTGTGCTGTACGG) directly upstream of the protospacer adjacent motif (Pa.m.). crRNAs were synthesized by Integrated DNA Technologies, Inc. (IDT) (Coralville, IA, USA). Alt-R^®^ CRISPR-Cas9 tracrRNA was also purchased from IDT. Each reagent was dissolved in Opti-MEM^®^ medium (#31985062; Thermo Fisher Scientific Inc., Waltham, MA, USA) at a final concentration of 100 μM, and stored at −80 °C until use. RNP complex was mixed with 0.5 μg/μL Cas9 protein (IDT), 30 μM dual gRNA (crRNA and tracrRNA), 0.02% Fast Green FCF (#15939-54; Nacalai Tesque Inc., Kyoto, Japan) dissolved in Opti-MEM^®^ medium. This mixture was hereinafter defined as “RNP + dye”. RNP complex with HA (#H3506; Merck KGaA, Darmstadt, Germany) was made by mixing RNP complex with 0.01% HA and 0.02% Fast Green FCF. This mixture was hereinafter defined as “RNP + HA + dye”.

For monitoring fluorescence in zygotes after *i*-GONAD-mediated delivery, a solution was prepared by mixing 1 mg/mL of RFD (#D3307; Thermo Fisher Scientific Inc.), 5 μg/mL Ho33342 (#4082; Cell Signaling Technology Inc., Danvers, MA, USA) and 0.02% Fast Green FCF in Opti-MEM^®^ medium, which is hereinafter defined as “RFD + Ho33342 + dye”. In some cases, a solution was prepared by mixing “RFD + Ho33342+ dye” with 0.01% HA, which is hereinafter defined as “RFD + Ho33342+ HA + dye”.

### 4.4. i-GONAD Procedure and Post-i-GONAD Treatment

*i*-GONAD was carried out at 10:30–11:00 a.m. (Days 0.4 of pregnancy), 13:00–13:30 p.m. (Days 0.5 of pregnancy) or 16:00–16:30 p.m. (Days 0.7 of pregnancy) on the day when vaginal plug was recognized after natural mating (Figure 1A). One to four pregnant females per session were used. Surgical procedures were performed on adult females anesthetized with a mixture of three anesthetic agents (medetomidine, 0.75 mg/kg; Nippon Zenyaku Kogyo Co., Ltd. Fukushima, Japan), midazolam (4 mg/kg; Sandoz K.K., Tokyo, Japan), and butorphanol (5 mg/kg; Meiji Seika Pharma Co., Ltd., Tokyo, Japan)) under a dissecting microscope, based on slightly modified versions of previously reported procedures [12]. The ovary/oviduct/uterus were exposed after making an incision in the dorsal skin and a subsequent incision in the muscle layer. The exposed ovary, oviduct, and part of the uterus were placed on the back skin of mice, and adipose tissue was anchored with an Aorta-Klemme to prevent return of the exposed tissues. Approximately 1.0 μL of solution was injected into the oviduct lumen upstream of the ampulla using a micropipette and an attached mouthpiece. Immediately (0 min) or 3 min after the injection, the oviduct regions were covered with a piece of wet KimWipe paper, soaked in Dulbecco’s modified phosphate buffered saline (DPBS), and grasped in tweezer-type electrodes. Electroporation was performed using a square-wave pulse generator, CUY21EDITII (BEX Co., Ltd. Tokyo, Japan). The electroporation parameters were as follows: PdA: 100 mA for B6 female or PdA: 250 mA for ICR female, Pd on: 5.00 ms, Pd off: 50 ms, pulse cycles: 3, Pd V: 50 V and decay: 10%. After in vivo EP, the oviducts were returned to their original position. The animals were then given an intradermal detoxicant, atipamezole (3.75 mg/kg; ANTISEDAN^®^, Nippon Zenyaku Kogyo Co., Ltd. Fukushima, Japan), and housed for further analysis.

In cases of *i*-GONAD using RFD + Ho33342 + dye or RFD + Ho33342 + HA + dye, the treated females were immediately sacrificed for fluorescence inspection, as shown below. In the case of *i*-GONAD using RNP (targeting *Tyr*) + dye, the treated females were allowed to survive until Day 12.5 of pregnancy for checking eye pigmentation and possible presence of indels at the *Tyr* locus. In the case of *i*-GONAD using RNP (targeting Gene A) + dye, pregnant females were allowed to deliver their pups for checking possible presence of indels at Gene A locus, as shown in Figure 1B.

### 4.5. Analysis of CRISPR/Cas9-Induced Indels

Tail biopsy was performed from the fetuses and pups for genotyping. Genomic DNA was isolated through incubation of the isolated samples in 100 μL of 50 mM NaOH at 95 °C for 10 min. Then, 10 μL of 1 M Tris-HCl (pH 8.0) was added to the aliquot and mixed. This crude DNA extract was used as a template for PCR. 

PCR amplification of a sequence corresponding to *Tyr* and Gene A were performed in a volume of 20 µL containing 10 µL of 2× PCR buffer for KOD FX (#KFX-101; TOYOBO, Osaka, Japan), 0.4 mM dNTPs, 1 µL of crude lysate, 0.25 µM primer pairs (Tyr–F: 5′-GGG GAG TGG TTA TAT AGG TC-3′/Tyr–R: 5′-TCT CGA ATT TCT TGT TCC CA-3′ for Tyr and Gene A-F: 5′-GTT CAT AAA GCA CTA AGG TC-3′/Gene A-R: 5′-GTA GGA AAG GAC ACC TCA CA-3′ for Gene A), and 0.1 U KOD FX (TOYOBO) under cycling conditions of denaturation at 94 °C for 3 min; amplification with 33 cycles of 95 °C for 20 s, 57 °C for 30 s, and 68 °C for 1 min; and extension at 68 °C for 5 min. Amplification products (5 μL) were separated by 2% agarose gel electrophoresis.

First, 5 µL PCR products were incubated with 1 µL Exonuclease I (#M0293; New England Biolabs, Inc., MA, USA) and 1 µL shrimp alkaline phosphatase (#M0371; New England Biolabs, Inc.) at 37 °C for 15 min, and then incubated at 80 °C for 15 min to inactivate both enzymes prior to addition of 90 µL sterile distilled water. Sequencing analysis was performed using the enzyme-treated PCR products and the primer Tyr–F or Gene A-F with a BigDye Terminator v3.1 Cycle Sequencing kit (Thermo Fisher Scientific Inc.) and then analyzed on an automated ABI PRISM 3100 DNA sequencer (Thermo Fisher Scientific Inc.). 

### 4.6. Fluorescence Analysis

Three min after *i*-GONAD with FRD + Ho33342 + dye or FRD + Ho33342 + HA + dye, oviducts were immediately dissected from the *i*-GONAD-treated females and subjected to zygote isolation through flushing the oviduct with DPBS + 0.3% fetal bovine serum (FBS). The isolated cumulus–zygote complex or zygotes were transferred to a drop (30 μL) of DPBS + 0.3% FBS. After covering the drop with a coverglass, fluorescence in the isolated cumulus–zygote complex or zygotes was assessed using ECLIPSE Ts2R fluorescence microscopic system and photographed (Nikon Co., Tokyo, Japan). 

### 4.7. Statistical Analysis

Statistical analysis of fetuses with di-pigmented eyes was performed using chi-square test. The *p* value for the chi-square test statistic was calculated in Excel.

## 5. Conclusions

The *i*-GONAD-based genome editing at early zygote stage can be greatly improved by delaying the timing of in vivo EP after intraoviductal instillation of RNP-containing solution, when compared with the previous method (in vivo EP immediately after intraoviductal instillation). Addition of HA to the RNP-containing solution is also beneficial for increasing the rate of indels. This simple modified protocol will soon be applicable when genome editing targeting early zygotes is required with relatively low costs and minimal equipment setup. 

## Figures and Tables

**Table 1:** *i-*GONAD ^1^ targeting Gene A at Day 0.5 of pregnancy (13:00 p.m.) using C57BL/6J mice.

Target Locus	Treatment	No. Mice Treated	No. Pregnant Mice	No. Pups (A)	No. Indels (B)	% (B/A)
Gene A	0 min with RNP + dye	7	3	13	3	23
	3 min with RNP + HA + dye	6	3	13	6	46

^1^*i*-GONAD was performed using RNP (targeting Gene A) + dye (0.02% Fast Green FCF) or RNP + hyaluronidase (HA) + dye. In the former case, in vivo electroporation (EP) was carried out immediately (0 min) after intraoviductal instillation of RNP + dye. In the latter case, in vivo EP was carried out 3 min after intraoviductal instillation of RNP + HA + dye. These treated females were allowed to survive for delivery of their pups.

## Data Availability

Not applicable.

## Supplementary Materials

The following supporting information can be downloaded at: https://www.mdpi.com/article/10.3390/ijms231810678/s1.

## Abbreviations

CRISPR/Cas9	clustered regularly interspaced short palindromic repeat-associated protein 9
ET	egg transfer
*Fgf10*	fibroblast growth factor 10
GM	genetically modified
*i*-GONAD	improved genome editing via oviductal nucleic acid delivery
Indel	insertion and deletion mutation
HA	hyaluronidase
Ho33342	Hoechst33342
KI	knock-in
KO	knock-out
Pa.m.	protospacer adjacent motif
PCR	Polymerase chain reaction
RFD	tetramethylrhodamine-labeled dextran 3 kDa
tracrRNA	trans-activating cr RNA
*Tyr*	tyrosinase
