# Supplementary material for: Successful i-GONAD in Mice at Early Zygote Stage through In Vivo Electroporation Three Min after Intraoviductal Instillation of CRISPR-Ribonucleoprotein
Source: Int J Mol Sci. 2022 Sep 14;23(18):10678. doi: 10.3390/ijms231810678 (PMC9506528; doi:10.3390/ijms231810678)
Supplement: Supplementary file 1 [file ijms-23-10678-s001.zip › ijms-1894915-supplementary.pdf]

Table S1. Summary of the *i*-GONAD-mediated genome editing targeting tyrosinase gene (*Tyr*) in the fetal offspring obtained after mating between female ICR and male C57BL6/J (B6) mice.

Time of *i*-GONAD[1]	Treatment[2]	No. pregnant mice/no. mice treated	No. fetuses obtained	No. fetuses carrying de-pigmented eyes (%)[3]	Fetuses with modified alleles[4]	
					No. indels (%)	No. mosaic alleles (%)
10:30 AM	0 min with RNP + dye	5/13	17	2 (12%)	3 (18%)	1 (6%)
	3 min with RNP + dye	3/11	20	11 (55%)	11 (55%)	4 (20%)
	3 min with RNP + HA + dye	5/6	20	14 (70%)	17 (85%)	6 (30%)
13:00 PM	0 min with RNP + dye	6/7	33	6 (18%)	6 (18%)	3 (9%)
	3 min with RNP + dye	6/7	30	21 (70%)	21 (70%)	9 (30%)
	3 min with RNP + HA + dye	5/6	19	15 (79%)	15 (79%)	4 (21%)
16:00 PM	0 min with RNP + dye	7/10	44	21 (47 %)	22 (50%)	9 (20%)
	3 min with RNP + dye	3/5	27	6 (22%)	12 (44%)	8 (30%)
	3 min with RNP + HA + dye	4/6	23	10 (43%)	13 (57%)	5 (22%)

[1]*i*-GONAD was performed on 10:30-11:00 AM (which is shown as "10:30 AM"), 13:00-13:30 PM (which is shown as "13:00 PM") or 16:00-16:30 PM (which is shown as "16:00 PM").

[2]*i*-GONAD was performed using RNP (targeting *Tyr*) + dye (0.02% Fast Green FCF) or RNP + hyaluronidase (HA) + dye. In the former case, *in vivo* electroporation (EP) was carried out immediately (0 min) or 3 min after intraoviductal instillation of RNP + dye. In the latter case, *in vivo* EP was carried out 3 min after intraoviductal instillation of RNP + HA + dye. These treated females were allowed to survive until Day 12.5 of pregnancy.

[3]Fetuses at Day 12.5 of pregnancy obtained after *i*-GONAD were first subjected to visual inspection for the presence of pigmented eyes. The fetuses having de-pigmented eyes are condidered genome-edited fetuses, since the endogenous wild-type *Tyr* locus in one allele derived form B6 males has been disrupted.

[4]Genotyping of tails from Day 12.5 fetuses obtained after *i*-GONAD was carried out. Direct sequencing of the PCR products revelaed that the samples have indels in non-mosaic or mosaic fashion.
